# Cocaine Tolerance in Honey Bees

**DOI:** 10.1371/journal.pone.0064920

**Published:** 2013-05-31

**Authors:** Eirik Søvik, Jennifer L. Cornish, Andrew B. Barron

**Affiliations:** 1 Department of Biological Sciences, Macquarie University, Sydney, Australia; 2 Department of Psychology, Macquarie University, Sydney, Australia; AgroParisTech, France

## Abstract

Increasingly invertebrates are being used to investigate the molecular and cellular effects of drugs of abuse to explore basic mechanisms of addiction. However, in mammals the principle factors contributing to addiction are long-term adaptive responses to repeated drug use. Here we examined whether adaptive responses to cocaine are also seen in invertebrates using the honey bee model system. Repeated topical treatment with a low dose of cocaine rendered bees resistant to the deleterious motor effects of a higher cocaine dose, indicating the development of physiological tolerance to cocaine in bees. Cocaine inhibits biogenic amine reuptake transporters, but neither acute nor repeated cocaine treatments caused measurable changes in levels of biogenic amines measured in whole bee brains. Our data show clear short and long-term behavioural responses of bees to cocaine administration, but caution that, despite the small size of the bee brain, measures of biogenic amines conducted at the whole-brain level may not reveal neurochemical effects of the drug.

## Introduction

Most research into the biology of addiction is done using animal models, of which the majority are mammalian, but increasingly invertebrate model systems are proving to be valuable for studying addiction related behavioural changes [1]–[3]. Recently *Caenorhabditis elegans*, *Drosophila*, and honey bees (*Apis mellifera*) have been used to study effects of drugs of abuse on the brain [1]–[4].

In many areas of neuroscience, use of simple invertebrate animal models has accelerated discovery of diverse neurobiological mechanisms underlying behaviour. Examples include the use of *Aplysia* to elucidate basic molecular mechanisms of learning and memory [5], and the molecular basis of circadian rhythms in *Drosophila* [6]. Recently invertebrate models have been introduced to addiction research. *C. elegans* has been used to study the molecular targets of drugs of abuse and drug induced modulation of neural plasticity [3]. *Drosophila* has been used most broadly in both cocaine and alcohol research. *Drosophila* demonstrate tolerance [7] and hedonic responses to ethanol [1], [8] and sensitisation to cocaine [9], [10]. Flies have also been used to examine the molecular effects of cocaine on the brain including particularly the importance of circadian genes [11] and transcription regulating LMO proteins in the development of cocaine sensitization [12], [13].

The honey bee has been used to study the effects of ethanol induced aggression [14]–[16], and effects of ethanol on decision-making [17], learning and memory [18], and ethanol reward in foraging preference [19], [20] and self-administration paradigms [21]. Honey bees have rich behavioural repertoires, and the work with ethanol has shown how it is possible to study many of the behavioural complexities associated with drug use in this simple invertebrate. Barron and colleagues [4] have shown that cocaine affects reward pathways in the bee brain, making the bee an ideal candidate model system for exploring the molecular effects of psychostimulants.

Addiction related behavioural changes are largely the result of neurobiological adaptations [22]. Therefore, for invertebrates to have utility for addiction research, it is important to assess to what extent short-lived organisms show long-term adaptation to repeated drug treatment. Here we further develop the bee as a model for addiction research by examining the effects of repeated cocaine administration on behaviour.

In rodents, repeated cocaine treatment leads to development of either sensitisation or tolerance, depending on the response studied, schedule, dose, and method of cocaine delivery [23]–[26]. Pharmacokinetics appear to be a critical factor in determining whether or not sensitisation takes place [26], since the same dose of cocaine can lead to different outcomes (tolerance versus sensitisation), depending on rate of delivery to the central nervous system [26]–[28]. Rapid drug delivery to central nervous systems (CNS) promotes development of sensitisation by altering the neurobiological impact of cocaine as well as inducing drug dependent brain plasticity [27].

In *Drosophila* sensitisation to cocaine was seen following a single treatment with volatilised [9] and injected cocaine [10]. There are, however, no reports of invertebrates developing tolerance following exposure to cocaine, or any other psychostimulants. This may occur, in part, due to the rapid delivery of the drug to the central nervous system following injection or volatilisation. In contrast, the method of cocaine delivery used thus far with honey bees is a topical application using dimethylformamide (DMF) as a solvent [4]. The benefit of this method is that it is largely non-invasive, permitting its use with free-flying animals still operating within their natural colony and foraging context. However, delivery with this method is reasonably slow, as cocaine must diffuse through the cuticle and then through the haemolymph before accessing the brain. We predicted that repeated topical treatment with cocaine to bees may result in the development of cocaine tolerance, given that extended and slow-delivery of cocaine produces tolerance in mammals [29], [30].

The immediate molecular targets of cocaine are biogenic amine (BA) re-uptake transporters. This is true for both mammals [31], and invertebrates [32]–[36]. The behavioural effects seen in response to cocaine administration in invertebrates are dependent on BA. In honey bees, effects of cocaine on reward perception can be mimicked by the BA octopamine (OA) [4], and in *Drosophila* cocaine sensitivity is affected based on brain dopamine (DA) levels [37], and tyramine is essential for the formation of sensitisation [38]. These effects are probably due to an increase in extracellular BA due to blockage of BA re-uptake transport. There is some variation in the affinity of cocaine for different specific BA transporters between species [31]–[36], but the general mode of action remains the same [31]–[36]. In mammals cocaine changes BA amounts in sub-regions of the brain following cocaine exposure [39], affects the turnover rate of dopamine [40], and has been seen to directly increase extracellular dopamine levels [41]. Following release BA are either recycled into the presynaptic cell [42] or metabolised into various breakdown products [43]. Blocking BA re-uptake transporters may therefore increase the proportion of released biogenic amines that are metabolised rather than recycled and thereby change the overall detectable levels of BA in the brain [44]. With a small nervous system (such as that of a bee) it is possible to quantify the impact of cocaine on total amounts of BA in the whole brain. Previous studies have linked changes in behavioural roles [45], foraging preferences [46], dance behaviour [47], aging [48], and stress [49] to changes in levels of BA measured at the whole-brain level, showing that this level of anatomical resolution is sufficient to detect behaviour-related changes in amine systems in insects. Here we examined the behavioural effects of repeated cocaine treatments to bees to determine what, if any, long-term neuroadaptive responses bees showed in response to repeated psychostimulant exposure. As a first step to exploring the physiological effects of cocaine on the bee brain, we assessed the impact of acute and chronic cocaine treatments on brain BA levels.

## Materials and Methods

### Subjects

European honey bees (*Apis mellifera*) were used for all experiments. Bees were raised in standard commercial hives housed at Macquarie University, Sydney, Australia, and kept according to standard beekeeping practices. For repeated treatment experiments, bees were housed in a 250 m^2^ flight enclosure to ensure that the only available food source was the feeder at which they were treated.

### Cocaine Treatment

For topical treatments, cocaine was dissolved in 1 µL of DMF and administered to the dorsal thorax of bees, using a 1 µL glass microcapillary pipette [4], [50]. This method is effective for delivering pharmacological agents to the brain of forager bees in a free-flight situation [4], [50]. As with all systematic treatments, there is some variation in total amount reaching the brain [50], but variation observed with this method is no larger than what is seen following intravenous and intraperitoneal cocaine administration in rats [51].

For treatment with volatilised cocaine, the method of McClung and Hirsh [9] was adapted for use with bees. Free-base cocaine was dissolved in 2 µL ethanol and applied to a nichrome wire filament. The ethanol was allowed to fully evaporate at room temperature (less than five min) to precipitate cocaine onto the filament. To sublimate the cocaine, the filament was heated by an electric current for 10 s. Electric current was applied such that the filament was heated to 200°C within 5 s, but did not surpass 350°C by 10 s. This heating profile allows for maximum sublimation of free-base cocaine, without the formation of methylecgonidine [52]. Cocaine was sublimated into an enclosed 50 cm^3^ chamber containing a single bee at a time. In this way bees were exposed to cocaine vapour for 1 min.

### Experiment 1: Effects of Repeated Cocaine Treatment on the Righting Reflex in Response to a High Cocaine Dose

The aim of this experiment was to determine whether repeated treatment with 3 µg of cocaine rendered bees more or less sensitive to the incapacitating effects of a higher cocaine dose. A 3 µg dose of cocaine delivered topically to a honey bee does not disrupt normal foraging or affect locomotion [4], however higher doses are known to be incapacitating to insects [9], [53]. To establish a suitable cocaine “challenge” dose, 50 free-flying foragers were treated at a sucrose feeder with either 10 or 20 µg of cocaine delivered topically to the thorax, the control group was treated with DMF only (vehicle control). Immediately post treatment bees were captured in a 50 mL specimen jar and observed for 150 min. No food was provided during the observation phase. The time-point at which the righting reflex [54] was lost, when bees lost coordination (usually fell to their backs and could not get up), was recorded for each bee.

**Figure 1:**
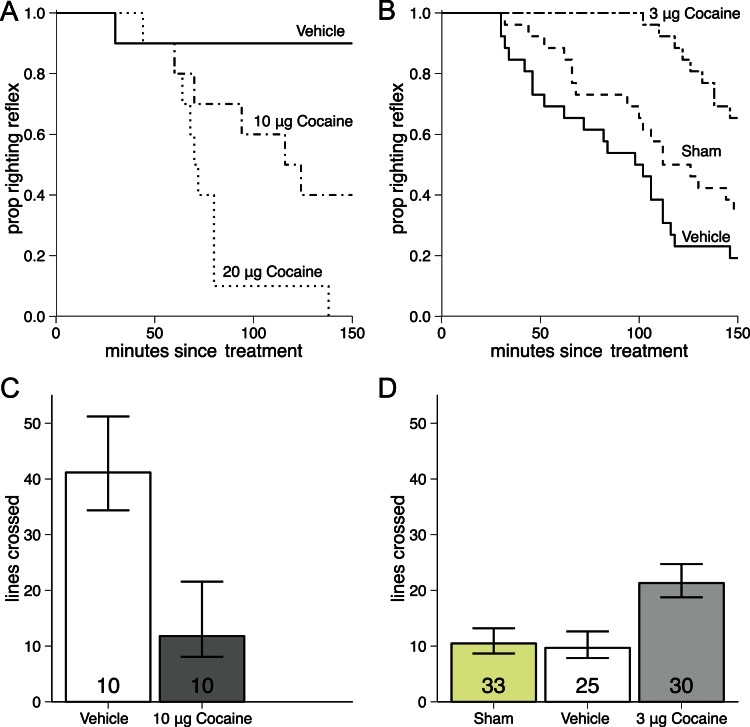
Survival plot of time to loss of righting reflex, and barplot of amount of locomotion for bees acutely and repeatedly treated with cocaine. Numbers indicate sample size per group. A. Survival plot of time to loss of righting reflex after acute treatment with vehicle control, 10 µg of cocaine, or 20 µg cocaine. The loss of righting reflex to cocaine administration occurred significantly earlier in cocaine treated groups than controls (Survival analysis: Kaplan-meier log-rank test: χ^ 2^ = 32.2, p<0.001, n = 10 per cocaine group; n = 30 for vehicle control). Pair-wise comparison showed that all groups differed from each another (10 µg vs DMF: χ^ 2^ = 10.5, p = 0.0012; 20 µg vs DMF: χ^ 2^ = 30.1, p<0.0001; 10 µg vs 20 µg: χ^ 2^ = 4.9, p = 0.0262). B. Survival plot of time to loss of righting reflex after being treated with 10 µg of cocaine following seven pretreatments with either 3 µg of cocaine, vehicle- or sham control. Time to loss of the righting reflex after administration of 10 µg cocaine was delayed in bees repeatedly pretreated with 3 µg of cocaine, compared to controls never treated with cocaine prior to the challenge dose (Survival analysis: Kaplain-meier log-rank test: χ^ 2^ = 18.4, p = 0.0001, n = 26 per group). Pair-wise comparison showed that both controls differed from cocaine treated bees (Sham: χ^ 2^ = 7.5, p = 0.0060; DMF: χ^ 2^ = 18.9, p<0.0001). Control groups did not differ from each other (Sham vs DMF: χ^ 2^ = 2.5, p = 0.1110). C. Amount of locomotion twenty mins after administration of vehicle control or 10 µg cocaine. Locomotion is reduced by a large cocaine dose (Mann-Whitney test: U = 22, p = 0.037). D. Amount of locomotion twenty min after administration of 10 µg cocaine after seven repeated treatments with 3 µg cocaine, or sham or vehicle control. The bees pretreated with 3 µg moved significantly after treatment with a 10 µg cocaine dose than the controls never treated with cocaine (ANOVA: F_2,85_ = 4.834, p = 0.011; Tukey’s multiple comparison between controls and cocaine: p = 0.012, between controls: p = 0.969).

Based on the results of the above experiment (Fig. 1A), 10 µg of cocaine was chosen as the challenge dose, since about half the bees had lost the righting reflex by the end of the 150 min observation period, compared to all bees in the 20 µg group. We therefore considered the 10 µg treatment more suitable for detection of either tolerance or sensitisation.

**Figure 2:**
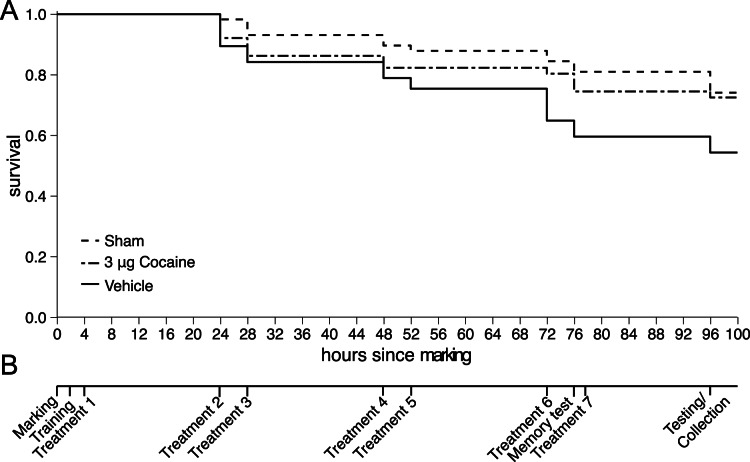
Treatment schedule and survival for repeatedly treated foragers. A. Survival curves for repeated treatment groups. Cocaine, vehicle control, and sham treated groups contained 51, 57, and 58 bees, respectively. Bees disappearing between marking and first treatment have not been included. Cocaine treated bees did not differ from vehicle (Kaplan-meier log-rank test: χ^ 2^ = 0.1, p = 0.750) nor sham control (Kaplan-meier log-rank test: χ^ 2^ = 3.4, p = 0.064), but there was a significant difference between the two control groups (Kaplan-meier log-rank test: χ^ 2^ = 5.4, p = 0.02). B. The schedule of treatments used in the repeated treatment experiments.

To assess how repeated 3 µg cocaine administration affected the bees response to 10 µg cocaine, bees were treated with seven 3 µg cocaine treatments over four days, before exposing them to the 10 µg dose. The experiment ran over a five-day period (Fig. 2B). The typical life-span of a foraging bee is approximately seven days [55], so the five-day duration of the experiment represents a significant portion of the bees foraging life-span. While feeding at a 1 M sucrose feeder foragers were individually marked with enamel paint on the morning of day one. That afternoon marked bees were treated with either 3 µg cocaine, vehicle control (DMF only), or touched on the thorax with an empty glass microcapillary pipette (sham treatment). For the next three days bees were treated in the same way twice a day, at least four hours apart. On the morning of the fifth day all bees were treated with 10 µg cocaine, captured and observed as above. Prior to the 10 µg challenge dose only the 3 µg cocaine treated group had previously been treated with cocaine, the other two groups were drug naïve. Throughout the treatment period, bees continued working inside and outside their hive in their natural social context. A total of 78 bees were sampled, evenly distributed across treatment groups.

### Experiment 2: Effects of Repeated Cocaine Treatment on the Locomotor Response to a 10 µg Cocaine Dose and long-term Memory Performance

Experiment 1 indicated cocaine pre-treatment rendered bees tolerant to the incapacitating effects of 10 µg cocaine. Experiment 2 examined how repeated treatments with a 3 µg cocaine affected bees’ locomotor responses to cocaine. In mammals cocaine is often described as a behaviour and motor stimulant, however at high doses cocaine causes reduced locomotion, and motor abnormalities in both insects [9], [53] and mammals [56], [57].

To establish the effects of 10 µg cocaine on bee locomotion, bees were treated and captured in the same way as in experiment 1. A total of twenty bees were used for this experiment: 10 were treated with cocaine and 10 with vehicle control. Twenty min after the drug treatment, bees were transferred to a 9 cm petri dish. The dish was placed on top of a 3 cm square-grid, and for 60 s the number of times a bee crossed a grid line was counted to provide an index of locomotor activity. Twenty minutes after treatment was chosen because at this time point none of the bees in experiment 1 had shown any gross behavioural defects. To examine the effect of repeated cocaine treatment of the locomotor response of bees, one hundred and eighty naïve bees (sixty per treatment group) were given the same schedule of seven 3 µg cocaine, vehicle, or sham treatments as in experiment 1 prior to testing locomotor performance after the challenge dose. Therefore at the point of treatment with the 10 µg cocaine challenge dose while the 3 µg cocaine group had been exposed to cocaine for four days, the two control groups were drug naïve, and the 10 µg cocaine challenge dose was the first exposure to cocaine for these bees. Because several bees died during the duration of the treatment schedule, final numbers varied slightly between groups.

To determine if either 3 µg cocaine or DMF had any harmful effects when given repeatedly to bees, survival of individual bees were estimated during the treatment period. The time point at which a bee was last seen at a feeder was used as an estimation of its time of death.

In addition, we also tested how repeated treatment affected retention of food related memory. The same bees were given a simple learning task on day one, prior to the first treatment. Two feeders were presented sequentially: a blue feeder containing 2 M sucrose, and a yellow feeder containing water. The feeders were presented for 10 min at a time. Bees received four exposures to each feeder. On day four, following the last treatment, both feeders were presented simultaneously. At this time both were empty. The first feeder each marked bee alighted on was recorded. The proportion of bees that chose the blue feeder on first landing was compared between treatment groups.

### Experiment 3: Effects of Acute and Repeated Cocaine Treatment on Biogenic Amine Levels in Honey Bee Brains

Cocaine is an antagonist of BA re-uptake transporters [35], [58] and would reduce efficiency of BA recycling once released. Here we examined if BA levels in honey bee brains were affected by cocaine treatment.

To assess the effects of acute cocaine treatment on BA levels, adult foragers were treated topically with 10 or 20 µg cocaine, or vehicle or sham controls as in experiment 1. Eighteen bees were allocated to each of the four treatment groups. Treated bees were flash-frozen in liquid nitrogen 2 hours after treatment, and BA levels were quantified by high-pressure liquid chromatography. The time-point choice was based on a pilot experiment with day old bees sampling at both 1 and 2 h after treatment (Fig. S1 in File S1). Based on these data we saw the greatest changes 2 h after treatment, and therefore used this time point when sampling forager bees following topical cocaine treatment.

To assess the effect of repeated treatment on BA levels, fifty bees were treated with seven 3 µg cocaine doses, vehicle or sham control treatments following the same schedule as in experiment 1 and 2 (Fig. 2B). Bees were flash frozen in liquid nitrogen in the morning of day five at the same time-point as used for the challenge dose and bioassay in experiment 1 and 2, and BA levels were quantified.

### Experiment 4: Effects of Volatilised Cocaine on Biogenic Amine Levels

All cocaine treatments used in the previous experiments were delivered in 1 µl of DMF, therefore, we wanted to remove any possible confounding effects of DMF administration on BA levels. Forty bees were treated with 5, 25, 50, 100, or 200 µg of volatilised freebase cocaine as per the method above. As a control, eight bees were exposed to a clean heated filament, from which pure ethanol had been fully evaporated. One hour after treatment bees were flash frozen in liquid nitrogen and brain BA content was analysed. We chose to freeze bees treated with volatilised cocaine 1 h after treatment, rather than 2 h as for topical treatment, as a more rapid absorption of cocaine was expected following this treatment method. At this point bees still differed significantly from controls in terms of their locomotion behaviour (see results), and whole brain amine levels have been shown to be affected 1 h after application of physical stressors [59], and similar time points have been used routinely in rodent studies [60], [61].

To compare bee behavioural responses to the novel volatilised cocaine exposure to the most commonly used topical application method, the locomotor assay above (Expt 2) was modified slightly. To see how the volatilised treatment affected the bees over time we placed the bees in the dish immediately following treatment and quantified locomotion for 60 s every 10 min. Using this method we compared locomotor effects of administration with 100 µg volatilised cocaine with controls. For this experiment fifteen bees were treated with 100 µg of volatilised cocaine, and fifteen bees were treated with control. We chose 100 µg for our volatilised treatments as this dose has previously been shown to be effective in eliciting behavioural responses in *Drosophila*, and bees treated with 10 µg volatilised cocaine did not show any of the gross behavioural defects seen following 10 µg topical treatment (ES personal observation).

### Biogenic Amine Analysis by High-Pressure Liquid Chromatography (HPLC)

Serotonin (5HT), DA, and OA were quantified by an Agilent 1200 Series HPLC system (Agilent Technologies, Santa Clare, CA, USA) coupled to an ESA Coulechem III electrochemical detector connected to an ESA 5011A high-sensitivity dual-electrode analytical cell (ESA, Chelmsford, MA, USA). Samples were separated across a 100 mm Thermo Fisher Scientific Hypersil 5 µm octadecylsilane packaged column (Thermo Fisher Scientific, Waltham, MA, USA).

Sample preparation followed the protocol of Barron and Robinson [62]. In brief, frozen bee brains were partially lyophilised [45], dissected while frozen over dry ice and immediately stored at −80°C until processing. Dissected brains included the central brain, antennal lobes, both optic lobes, and the suboesophageal ganglion, but no occeli [63]. Frozen brains were centrifuged at 15 G for 5 min, after which brains were homogenised by sonication in 60 µl of 0.2 M perchloric acid containing 10 pg/µL dihydroxybenzylamine (DHBA) as an internal standard. Homogenised brains were incubated on ice in darkness for 20 min, before being centrifuged at 15 G for 15 min. For each sample 10 µL of the supernatant was analysed.

BA amounts were quantified relative to a standard curve created by injecting seven successive perchloric acid solutions containing 10 pg/µL of DHBA and varying amounts of DA, OA, and 5HT. The solutions were created in decreasing steps, so that the first injection contained of 10 pg/µL DA and 5HT, and 5 pg/µL OA, and each successive injection had 1.5 pg/µL less DA and 5HT and 0.75 pg/µL less OA than the previous injection. The seventh injection contained only DHBA. For each BA, a linear regression of the peak area relative to the area of the DHBA peak was fitted against the known BA quantity. The seven injections were repeated after every 24 samples, and samples were quantified relative to the averages of the two standard curves run before and after each set of samples. In each batch of 24, samples from all experimental groups in an experiment were block-randomised. Standards were created from frozen stocks, and prepared on the day of each run. All compounds used for making the standard curve were supplied by Sigma-Aldrich (St. Louis, MO, USA).

### Statistical Analysis

All analysis were carried out using R version 2.15.0 [64]. Survival analysis in experiment 1 and 2 was performed using Kaplan-meier log-rank tests. For each experiment all curves were compared together, followed by pair-wise comparisons of relevant pairs. For experiment 3 and 4, BA levels were analysed using a Multivariate Analysis of Variance (MANOVA) procedure. The amounts of OA, DA, and 5HT were fitted against the treatment group. This allows for comparing the effects of the treatments on each biogenic amine, as well as the interrelatedness between the biogenic amines.

## Results

### Experiment 1: Effects of Repeated Cocaine Treatment on the Righting Reflex in Response to a High Cocaine Dose

Acute cocaine treatment with a high dose caused loss of righting reflex in a dose-dependent manner. The majority of bees treated with 10 µg cocaine, and all of the 20 µg treated bees lost the righting reflex by the end of the experiment (Fig. 1A). Loss of righting reflex following cocaine administration occurred significantly earlier in cocaine treated groups compared to controls (Survival analysis: Kaplan-meier log-rank test: χ^2^ = 32.2, N = 50, p<0.001). Pair-wise comparison showed that both 10 and 20 µg cocaine treated bees were significantly different from vehicle controls (10 µg vs. DMF: χ^2^ = 10.5, p = 0.0012; 20 µg vs. DMF: χ^2^ = 30.1, p<0.0001), and the two cocaine groups differed from each other (10 µg vs. 20 µg: χ^2^ = 4.9, p = 0.0262). When analysing the effect of repeated treatment with cocaine on response to the 10 µg cocaine dose, cocaine pretreated bees remained mobile for significantly longer than both sham and vehicle pretreatment control groups (Fig. 1B, Kaplan-meier log-rank test: χ^2^ = 18.4, N = 78, p<0.001). Pair-wise comparison showed that both controls differed significantly from the cocaine treated groups (Sham: χ^2^ **= **7.5, p = 0.0060; DMF: χ^2^ = 18.9, p<0.0001), and the two control groups did not differ from each other (Sham vs DMF: χ^2^ = 2.5, p = 0.1110).

### Experiment 2: Effects of Repeated Cocaine Treatment on the Locomotor Response to a 10 µg Cocaine Dose and Long-term Memory Performance

Acute treatment with 10 µg cocaine significantly reduced locomotion relative to vehicle control (Mann-Whitney test: U = 22, N = 20, p = 0.037, Fig. 1C), non-parametric Mann-Whitney U test was used as the data was not normally distributed. However the effect of 10 µg cocaine on locomotion was counteracted by repeated pretreatment with 3 µg cocaine (Fig. 1D). Bees pretreated with 3 µg cocaine, sham or vehicle differed in their total amount of locomotion following treatment with 10 µg cocaine (One-Way ANOVA: F_2,85_ = 4.834, p = 0.011). Tukey’s multiple comparison test showed that the two control groups did not differ from each other (p = 0.969), but did differ from the 3 µg cocaine treatment (p = 0.012).

Bee survival differed significantly between the repeated treatment groups (Kaplan-meier log-rank test: χ^2^ = 6.5, N = 166, p = 0.0382, Fig. 2A), but pair-wise comparisons did not show any difference between the cocaine treated group and sham or vehicle controls (Kaplan-meier log-rank test: χ^2^ = 3.4, p = 0.064, Kaplan-meier log-rank test: χ^2^ = 0.1, p = 0.750, respectively), however there was a significant difference between the control groups (Kaplan-meier log-rank test: χ^2^ = 5.4, p = 0.02). Additionally, bee performance in a simple memory assay was uniformly high across cocaine, sham, and vehicle repeat treatment groups (χ^2^ = 1.238, N = 62, p = 0.5384, Fig. S2 in File S1) suggesting repeated cocaine treatments did not alter memory of a food reward (out of 62 bees, approx. 20 per group, only two bees made an incorrect choice, one cocaine- and one vehicle-treated).

### Experiment 3: Effects of Acute and Repeated Cocaine Treatment on Biogenic Amine Levels in Honey Bee Brains

**Figure 3:**
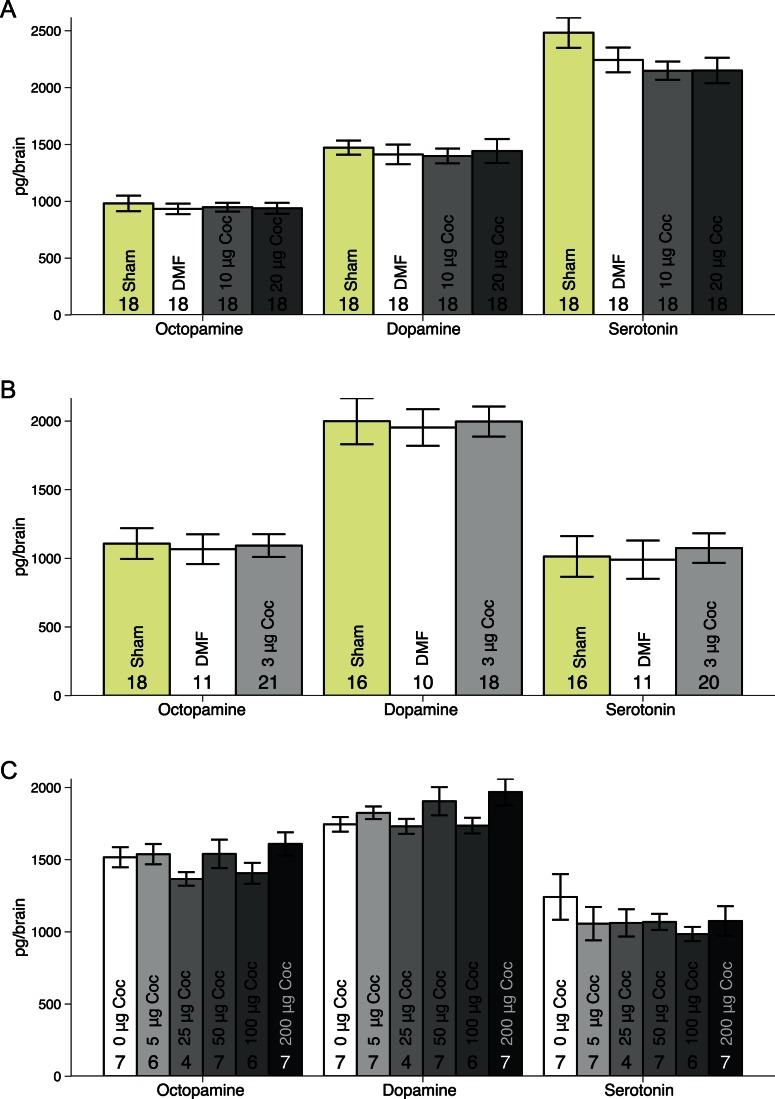
Amount of biogenic amines in whole brains of bees following acute or repeated cocaine treatment. A. Biogenic amine content of brains frozen two hours after being treated with sham, vehicle control, 10 µg or 20 µg cocaine. There were no significant differences between the groups (MANOVA: F_6,74_ = 0.2859, p = 0.9419). B. Biogenic amine content of brains treated seven times with sham, vehicle control, or 3 µg cocaine. No significant differences were seen between the groups (MANOVA: F_6,72_ = 0.3549, p = 0.905). C. Biogenic amine content of brains of bees frozen one hour after treatment with 0, 5, 25, 50, 100, or 200 µg of volatilised cocaine. There were no significant differences between the treatment groups (MANOVA: F_15,93_ = 0.9069, p = 0.5561, Fig. 3C).

OA, DA, and 5HT levels did not differ between bees treated acutely with 10 or 20 µg cocaine, or vehicle or sham controls (MANOVA: F_6,74_ = 0.2859, p = 0.9419, Fig. 3A), nor did levels of OA, DA, and 5HT differ significantly between groups receiving repeated treatments of 3 µg cocaine, sham or vehicle control (MANOVA: F_6,72_ = 0.3549, p = 0.905, Fig. 3B). Therefore we saw no evidence of acute or repeated topical cocaine treatment affecting levels of BA in the brains of honey bees. Previously, we have had similar results with day olds (Fig. S1 in File S1), and shorter time intervals (Fig. S3in File S1).

### Experiment 4: Effects of Volatilised Cocaine on Biogenic Amine Levels

**Figure 4:**
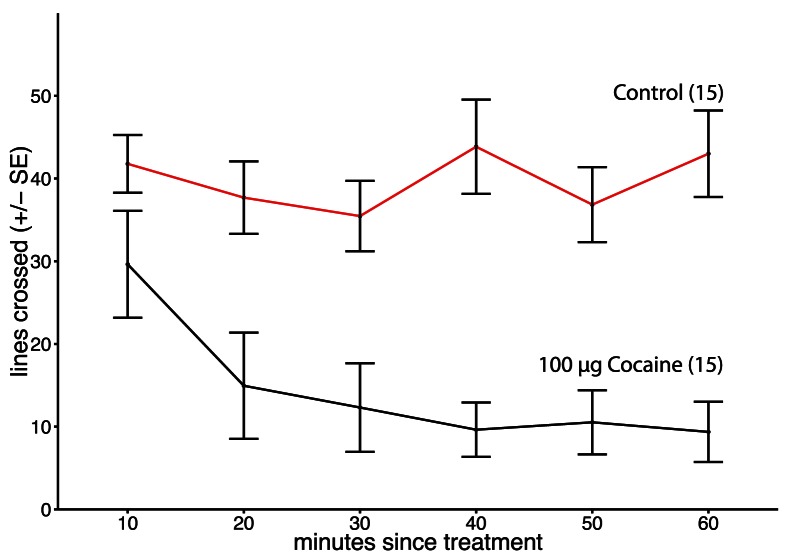
Locomotion over time in bees following volatilsed cocaine treatment. Locomotion was significantly reduced in bees treated with 100 µg of volatilised cocaine compared to controls (Repeated measures ANOVA: F_1,34_ = 23.998, p<0.001, Fig. 4), but was the same at all time intervals measured (Repeated measures ANOVA: F_1,34_ = 0.007, p = 0.934, Fig. 4).

Locomotion was significantly reduced in bees treated with 100 µg of volatilised cocaine compared to controls (Repeated measures ANOVA: F_1,34_ = 23.998, p<0.001, Fig. 4), but was the same at all time intervals measured (Repeated measures ANOVA: F_1,34_ = 0.007, p = 0.934, Fig. 4). Despite this behavioural effect, no change in OA, DA, or 5HT was detected following acute treatment with 5–200 µg volatilised cocaine (MANOVA: F_15,93_ = 0.9069, p = 0.5561, Fig. 3C).

## Discussion

Here we have demonstrated that in honey bees repeated topical cocaine treatment lead to development of tolerance to the detrimental effects of a large cocaine dose (Fig. 1). Repeated treatment with 3 µg cocaine reduced inhibition of locomotor activity by 10 µg cocaine, and reduced severity of the loss of coordination seen in bees following this high cocaine treatment (Fig. 1). Following seven repeated treatments with 3 µg cocaine, bees had less of a response to 10 µg of cocaine than drug naïve bees that had only been treated with vehicle or sham. Thus demonstrating that the effect of 10 µg cocaine was reduced by previous drug administration. Previously only sensitisation had been demonstrated following cocaine treatment in invertebrates: it has been seen in flies following treatment with volatilised delivery [9] and intra-abdominal injection [10] as well as following injection directly in to the head ganglion of crayfish [65]. Taken together, the previous invertebrates research, and our current findings, shows that both sensitisation and tolerance can in occur in response to psychostimulant administration in invertebrates. Despite these clear behavioural results we found no evidence of any cocaine treatment affecting BA levels in the whole bee brain.

In mammals [25], both tolerance and sensitisation can develop, depending on the rate of drug delivery to the CNS s(ug delivery livery ry (samaha)ependent on rate of drug delivery (samaha) and schedule.ted twice f the drug to the central nerv [26] and administration schedule [66]. One important factor determining whether sensitisation develops is delivery rate [26], with rapid delivery protocols being more likely to cause sensitisation [28]. The invertebrate experiments demonstrating sensitisation used treatment methods that rapidly delivered cocaine to the CNS, while the topical treatment method employed here was relatively slow to deliver cocaine to the CNS. This difference could clearly be seen in that a loss of the righting reflex was first seen 60 min after topical treatment with cocaine (Fig. 1A), but in bees treated with volatilised cocaine a similar effect was seen almost immediately after treatment (Fig. 4; ES personal observation).

The administration schedule also influences the development of sensitisation [67], with intermittent drug treatment schedules being more likely to cause sensitisation to cocaine [68]. Sensitisation was seen in crayfish when a single cocaine injection was given every day for three days [69]. This treatment regime has previously been effective in producing sensitisation in rats [70]. In *Drosophila* strongest sensitisation was seen after a single volatilised cocaine exposure 6 hours prior to the challenge dose [9]. Similarly, a single cocaine exposure has also been seen to cause sensitisation in rodents when the challenge was given 24 hours after the original dose [71]. Tolerance in mammals has been reported following continuous (chronic) cocaine treatment [23], [24]. Bees in our experiment were treated twice a day for four days. It is possible that the tolerance we observed in honey bees is a consequence of the slow delivery of cocaine caused by the tropical treatment method, combined with multiple treatments per day, effectively resulting in bees being given a near chronic cocaine treatment. Similar exposure regimes has previously been shown to produce tolerance in rodents [29], [30], [72].

It is possible that the schedules and delivery regimes generating tolerance and sensitisation in invertebrates may be broadly similar to those that give the same results in mammals. To test this it will be necessary to show that both sensitisation and tolerance can be induced in the same invertebrate species using different treatment regimes. Further work could examine this issue in honey bees using both the topical and volatilised treatment methods.

The tolerance seen here could have resulted from a change in the pharmacokinetic response to the drug administration, or a pharmacodynamic response to the cocaine, or both. Pharmacokinetic tolerance describes a change in the concentration of cocaine reaching the brain as a result of previous cocaine treatment. Pharmacodynamic tolerance results from a change in the effect of cocaine on the CNS.

Pharmacokinetic tolerance could occur as a result of how topically administered cocaine is absorbed or metabolised. Honey bees possess a wide-range of detoxification enzymes [73], and have been known to respond to toxins by increasing the activity of mixed function oxidases [74], which are involved in drug metabolism. In bees induction of detoxification enzymes is a slow process [74], but it is possible this may have contributed to the development of tolerance seen here. Measurement of the amount of cocaine reaching the bee brain would be needed to examine whether bees are becoming tolerant simply by increasing the metabolism and excretion of cocaine. Another possibility is that cocaine simply did not enter the brain following our treatments, and that our results are not due to effects of cocaine on the brain. The observed lack of change in brain BA levels could suggest this, but we find this unlikely for the following reasons: Topical treatment allow neuroactive compounds to enter honey bee brains [4], [50], and cocaine administered in this way affect complex behavioural responses suggestive of the involvement of the central brain [4]. Furthermore, the blood brain barrier is the same for the brain and peripheral ganglia [75], meaning that if cocaine is able to enter and effect ganglia, there is no reason to assume that it could not enter the brain as well. Further, we assume that treatment with volatilised cocaine would allow cocaine to diffuse through the tracheal system. Tracheoles terminate at neurons directly within the brain. These junctions are not protected by the insect blood-brain barrier and therefore this delivery method should give rapid access of cocaine to the nervous system. The rapid behavioural responses seen with this delivery method support such an interpretation.

Pharmacodynamic changes are considered the main cause of cocaine tolerance in mammals [76]. Examples of pharmacodynamics tolerance include reduction in the basal extracellular dopamine levels in the nucleus accumbens following chronic cocaine administration [77], as well as reduction in the quantity of dopamine that is released in response to a cocaine exposure [78]. This could be caused by inhibition of DA release due to increased D_2_-like autoreceptor sensitivity [79], or reduced availability of DA because of reduced synthesis [60], both of which have been seen following repeated cocaine exposure in rodents. In our study we found no change in brain BA levels following repeated topical cocaine treatment (Fig. 3B). Similarly acute topical (Fig. 3A) and volatilised (Fig. 3C) treatment methods did not cause measurable changes in whole brain BA levels either. As further support of these findings we observed no change in BA levels following acute treatment with 10 or 20 µg cocaine doses to day old bees after 1–2 hours (Fig. S1in File S1), or in forager bees with doses ranging from 3–30 µg cocaine after 30 min (Fig. S3in File S1). In mammals decreased synthesis of both DA and 5HT is seen in the nucleus accumbens, prefrontal cortex, piriform cortex, and the striatum following acute cocaine treatment [39]. A limitation of the method we used to quantify BA levels is that it does not distinguish between extra- and intracellular levels, and it is therefore possible that any increase or decrease in BA synthesis was masked by a corresponding change in BA turnover. Although examining whole brain levels of BA has undoubtedly been useful in the invertebrate neuroscience literature [45], [48], it is clear from these data, that when it comes to understanding drug action it is necessary to also consider the potential for altered BA turnover, and not just total amounts.

At this point it is unclear if the tolerance is due to pharmacokinetic or pharmacodynamic changes, or both. In order to distinguish if tolerance in bees is due to pharmacokinetic or pharmacodynamic changes it is necessary to examine changes in cocaine and BA metabolism. If cocaine metabolism increases with repeated treatment it would indicate that a pharmacokinetic change has taken place, conversely a change in BA metabolism would indicate pharmacodynamic tolerance. Unfortunately, the HPLC system utilised here was unable to detect BA metabolites, so we do not have any information on the turnover of these amines. This remains an open question for future studies.

To conclude, our data provide the first evidence for tolerance to a psychostimulant in an invertebrate, and thereby broaden the potential utility of invertebrates as models for the neurobiological effects associated with drugs of abuse. Our study also found that repeated cocaine treatment did not show changes to total BA levels, and highlights the need to consider the difference between extracellular and intracellular neurotransmitter levels when investigating mechanisms of drug action in invertebrate animals.

## Supporting Information

File S1 **This file includes Figures S1, S2 and S3.**
(DOCX)
