# Supplementary material for: Cocaine Tolerance in Honey Bees
Source: PLoS One. 2013 May 31;8(5):e64920. doi: 10.1371/journal.pone.0064920 (PMC3669089; doi:10.1371/journal.pone.0064920)
Supplement: File S1 — This file includes Figures S1, S2 and S3. (DOCX) [file pone.0064920.s001.docx]

**Supplemental Figs:**

**Figure S1**

**Figure S1.** Mean biogenic amine contents in the brains of day-old bees frozen one and two hours following topical treatment with either sham (yellow), vehicle control (white), 10 (light grey) or 20 (dark grey) μg of cocaine (error bars represent one standard error). **A.** There was no significant differences between bees treated with either 10 μg of cocaine, sham, or vehicle control one hour after administration (MANOVA: F_6,86_ = 2.0954, p = 0.6193). **B.** There were no significant differences between bees treated with either 20 μg of cocaine, sham, or vehicle control one hour after administration (MANOVA: F_6,94_ = 1.5286, p = 0.1773). **C.** There was a significant difference between bees treated with either 10 μg of cocaine, sham, or vehicle control two hours after administration (MANOVA: F_6,87_ = 2.9792, p = 0.0108), pair-wise comparisons showed the difference was between the control groups (p = 0.0013). **D.** There was a significant difference between bees treated with 20 μg of cocaine, sham, or vehicle control two hours after administration (MANOVA: F_6,66_ = 4.6928, p = 0.0005), however, using a bonferroni correction (α = 0.05/3) there was no significant difference between the DMF and the cocaine treated bees (p = 0.0331). There was a significant difference was between cocaine treated bees and sham treated bees (p = 0.0021), as well as between the DMF and sham treated bees (p = 0.0082). Indicating that this difference may be the result of the DMF application rather than an effect of cocaine.

**Figure S2**

**Figure S2.** Proportion of correct choices in a two-choice memory recall task following seven repeated treatments with either 3 μg cocaine, vehicle or sham controls. Performance was uniformly high (χ2 = 1.238, N = 62, p = 0.5384, Fig S1) suggesting that repeated cocaine or vehicle treatments did not affect memory performance.

**Figure S3**

**Figure S3.** Mean biogenic amine contents in the brains of forager bees flash-frozen 30 min after being treated topically with sham (yellow), vehicle control (white), or 3 (lightest grey), 9, 15, or 30 (darkest grey) μg of cocaine (error bars represents one standard error). There were no significant differences between the treatment groups (MANOVA: F_15,192_ = 1.632, p = 0.0684).

.
